# Evolution of the chitin synthase gene family correlates with fungal morphogenesis and adaption to ecological niches

**DOI:** 10.1038/srep44527

**Published:** 2017-03-16

**Authors:** Ran Liu, Chuan Xu, Qiangqiang Zhang, Shiyi Wang, Weiguo Fang

**Affiliations:** 1 Institute of Microbiology, College of Life Sciences, Zhejiang University, Hangzhou, 310058, Zhejiang, China; 2 Institute of Insect Sciences, Zhejiang University, Hangzhou 310058, Zhejiang, China

## Abstract

The fungal kingdom potentially has the most complex chitin synthase (CHS) gene family, but evolution of the fungal CHS gene family and its diversification to fulfill multiple functions remain to be elucidated. Here, we identified the full complement of CHSs from 231 fungal species. Using the largest dataset to date, we characterized the evolution of the fungal CHS gene family using phylogenetic and domain structure analysis. Gene duplication, domain recombination and accretion are major mechanisms underlying the diversification of the fungal CHS gene family, producing at least 7 CHS classes. Contraction of the CHS gene family is morphology-specific, with significant loss in unicellular fungi, whereas family expansion is lineage-specific with obvious expansion in early-diverging fungi. ClassV and ClassVII CHSs with the same domain structure were produced by the recruitment of domains PF00063 and PF08766 and subsequent duplications. Comparative analysis of their functions in multiple fungal species shows that the emergence of ClassV and ClassVII CHSs is important for the morphogenesis of filamentous fungi, development of pathogenicity in pathogenic fungi, and heat stress tolerance in Pezizomycotina fungi. This work reveals the evolution of the fungal CHS gene family, and its correlation with fungal morphogenesis and adaptation to ecological niches.

The fungal kingdom, with an estimated 1.5 million different species, displays extraordinary evolutionary diversity, which is reflected in different life histories, developmental processes and ecological niches^1^. Chitin is a linear polysaccharide that consists of N-acetylglucosamine (GlcNAc), and it is a key cell wall component of fungi, providing structure and protection for cells^2^,^3^. The general chitin synthesis pathway can be divided into 3 sequential reactions; the final reaction is catalyzed by chitin synthase (CHS), which is specifically associated with chitin biosynthesis^4^. All members of the CHS gene family possess the conserved region CON1, which contains the conserved motifs QXXEY, (E/D)DX, and Q(R/Q)XRW^5^,^6^. These 3 motifs are essential for catalytic activity^7^. CON1 also exists in the bacterial chitooligosaccharide synthase, NodCs^8^.

The fungal kingdom could be one of the eukaryotic groups with the most complex CHS gene family, but the number of CHSs varies significantly among fungi. One to 3 CHSs have been found in yeasts, whereas filamentous fungi usually have approximately 7 CHSs, with some species possessing more than 20 CHSs^9^. Using the conserved region CON1, a number of studies have investigated the evolution of fungal CHSs. When a small number of CHSs from dikarya fungi were used, fungal CHSs could be phylogenetically divided into 7 classes (I to VII) with high bootstrap support^5^,^10^,^11^,^12^,^13^. The 7 classes of CHS are usually grouped into 3 divisions, each with distinct domain structures^11^. Division 1 includes classes I, II and III, which contain PF08407 and PF01644 (CS1 domain). Division 2 contains classes IV, V and VII, which include PF00173, PF00063, PF03142 (CS2 domain) and PF08766. Division 3 only has class VI, which consist solely of PF03142. Recently, a phylogenetic analysis was conducted based on the CON1 region to investigate the evolution of a total of 347 CHSs in 54 genomes from dikarya and early-diverging fungi; these CHSs were divided into 2 divisions with high bootstrap support, but further classification was supported by low bootstrap values^6^. Similarly, based on 978 CHSs from 108 fungal species, Li *et al*. categorized the fungal CHSs into 8 classes using phylogenetic analyses, which had not been reported in previous studies investigating fungal CHSs^14^. They first classified the CHSs into 3 divisions by examining the presence of the CS1, CS2 and CS1N domains, and the CHSs within each division were then phylogenetically analyzed without outgroups. They categorized Division 1 into 4 classes, but class II was supported by a low bootstrap value. Likewise, class V was also poorly supported in the analysis of Division 2 and Division 3 CHSs^14^. Therefore, fungal CHSs have a complex, yet unresolved, evolutionary history, and new strategies are needed to analyze such histories when a large number of CHSs are considered.

A functional genomics conundrum concerns how a complex protein family such as the CHS gene family can diversify to fulfill multiple functions^15^. The functions of CHSs have been characterized in a number of fungi. CHSV and CHSVII, which contain an N-terminal myosin motor-like domain (MMD, PF00063), are important for morphogenesis by supporting hyphal tip growth in filamentous *Aspergillus nidulans, Colletrotrichum graminicola, Fusarium verticilliodes, Aspergillus fumigatus, Neurospora crassa* and *Ustilago maydis*^16^,^17^,^18^,^19^,^20^,^21^,^22^,^23^,^24^. However, other CHS classes show diverse biological functions among different fungal species. CHSI is related to morphogenesis in *Botrytis cinerea*^25^,^26^ but not in *A. nidulans* and *A. fumigatus*^27^,^28^. Characterization of the biological functions of CHSs in other fungi could provide additional information; with an understanding of the evolution of the fungal CHS gene family, information about the functions of CHSs could advance our knowledge regarding the relationship between CHS gene family evolution and diversification of the biological functions of CHS family members.

In this study, we identified the full complement of CHSs from 231 fungal species encompassing 9 fungal phyla. Using the largest dataset to date, we characterized the evolution of the fungal CHS family using phylogenetic and domain structure analyses. The biological functions of CHSs have been characterized in fungi that are pathogenic to mammal, plants and in many saprophytic fungi. In this study, we characterized the biological functions of CHSs in the model insect pathogenic fungus *Metarhizium robertsii*. By analyzing the biological functions of CHSs in multiple fungal species, we investigated the relationship between the evolution of the fungal CHS gene family and development of fungal pathogenicity, heat stress tolerance and morphogenesis.

## Results

### Phylogeny of fungal CHSs

Using an HMM (hidden Markov model) search, we retrieved putative CHSs from 231 fungal species encompassing 9 fungal phyla: Ascomycota, Basidiomycota, Glomeromycota, Zygomycota, Blastocladiomycota, Chytridiomycota, Neocallimastigomycota, Microsporidia, and Cryptomycota (Fig. S1, Table S1). False positives were removed by examining the presence of conserved domains and motifs of CHSs, resulting in the identification of 1,571CHSs (Fig. S1, Table S2) that included all CHSs identified in several previous studies^6^,^11^,^12^,^13^, suggesting that our method for retrieving CHSs from fungal genomes was effective.

**Figure 1:**
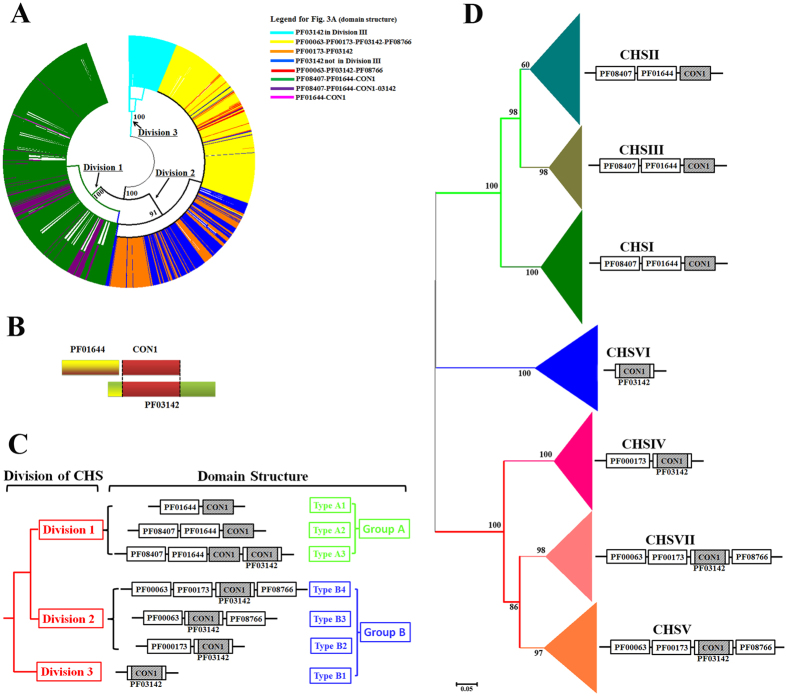
Phylogeny and domain structure of fungal CHSs. (**A**) The phylogenetic tree constructed using the protein sequence of the CON1 regions of all 1,571 fungal CHSs. The same color indicates proteins with the same domain structure. (**B**) Diagram showing the relative position of the CON1 region in domain PF03142 and the PF01644-CON1 region in Division 1 CHSs. (**C**) Types of domain structure of fungal CHSs and their distribution in the 3 divisions of fungal CHSs. The relative position of the CON1 region is shadowed. (**D**) The phylogenetic tree constructed with 832 Ascomycota CHSs. The 7 clades correspond to 7 classes of CHSs, respectively. In each clade, all proteins have the same domain structure, as shown on the right. Numbers at the nodes in the phylogenetic trees represent maximum-likelihood bootstrap values.

The CON1 region that is encompassed by the QXXEY, QXRRW, and EDRXL motifs was extracted from the 1,571 CHSs; the length of CON1 regions varied among fungi (Table S2). The length of the full CHS proteins ranges from 150aa (amino acids) to 2,172aa with an average length of 1,185aa; the minimum, maximum and average size of the CON1 regions is 97aa, 1,200aa and 141aa, respectively. All the 1,571 CHSs were used to generate a multiple alignment using the MUSCLE algorithm^29^, which was then used to construct an ML (Maximal likelihood) phylogenetic tree. This tree had 3 major clades with high bootstrap support values (≥91%), which were designated as Division 1 to 3, respectively (Fig. 1A). However, the subclades in the tree were supported by low bootstrap values (<50%). Thus, the evolution of fungal CHSs could not be resolved solely by phylogenetic analysis of the CON1 region. A previous study showed that the evolution of fungal CHSs could not be resolved by phylogenetic analysis using their full-length protein sequences^6^, so we did not perform this type of phylogenetic analysis.

The dominant evolutionary mechanisms for expansion of the protein repertoire are duplication, divergence, and recombination of the domains^30^. Thus, we combined the domain structure analysis with the phylogenetic analysis of CON1 sequences to investigate the evolution of CHSs. The domain structure analysis resulted in the identification of 20 domain types (Table S3). PF03142 (containing the CON1 region), PF00063, PF00173, PF08766, PF01644 and PF08407 were widely present in fungal CHSs; 14 other domains were only found in a very small number of fungal CHSs and did not display a clear distribution pattern. The domain structures of fungal CHSs could be divided into 2 groups (Fig. 1C, Table S2). Group A contains PF01644 (CS1) followed by the CON1 region (designated as PF01644-CON1 in this study) (Fig. 1B). Group A can be subclassified into 3 types; Type A1 consists only of PF01644-CON1, Type A2 contains PF08407- PF01644-CON1 and Type A3 consists of PF08407- PF01644-CON1 followed by PF03142. Group B contains PF03142 (CS2 domain) but not PF01644, and it can be subclassified into four major types; Type B1 contains only PF03142, Type B2 has PF03142 and PF00173, Type B3 consists of PF00063, PF03142 and PF08766 and Type B4 contains PF00063, PF00173, PF03142 and PF08766. Next, we mapped the domain structure of each protein to the above phylogenetic tree (Fig. 1A). Division 1 contained only CHSs with a Group A domain structure; CHSs with a Type A2 domain structure constituted the major part of the clade; and proteins with Type A1 and Type A3 domain structures were randomly scattered throughout the clade. CHSs with the Group B domain structure were present in Division 2 and Division 3. Division 2 contained most of the CHSs with Type B2 and Type B4 domain structures; CHSs with Type B1 and Type B3 domain structure were randomly scattered throughout this division. Division 3 only contained CHSs with the Type B1 domain structure.

Three hundred and ten CHSs had Type A1, A3, B1 or B3 domain structures and were randomly scattered throughout the phylogenetic tree of CON1 (Table S4); there was no consistency between their domain structure and phylogeny. There were 294 CHSs with a Type B1 domain structure, of which 95 were clustered in Division 3 (colored in light blue in Fig. 1A) and 199 were part of the 310 CHSs (colored in blue in Fig. 1A). Among the 199 CHSs with the Type B1 domain structure, 110 were from phylogenetically distant early diverging fungi, and 89 were mostly from Ascomycota or Basidiomycota yeasts. The 199 CHSs themselves were also not phylogenetically related, indicating that they could have resulted from independent domain duplications and were under different selection pressure. Fifty-one of the 310 CHSs had a Type A3 domain structure, but they were not phylogenetically related; 44 of the 51 CHSs were from yeasts, suggesting their association with the development of unicellular morphology. Therefore, these 310 CHSs could have a very complex evolutionary history that resulted in noise in the phylogenetic tree of all CHSs (Fig. 1A). To minimize such noise, their sequences were excluded (Table S4), and the CON1 regions of the remaining 1,261 CHSs were subjected to a phylogenetic analysis. The resulting trees also consisted of 3 divisions, which were subclassified into 4 clades with high bootstrap support (>80%) (Fig. S2, Table S5); the subclades were supported by low bootstrap values (<50%). In some previous reports^6^,^14^, evolution of fungal CHSs was not well characterized with low bootstrap support when CHSs from multiple phyla were used. On the other hand, we found that only Ascomycota fungi had CHSs distributed in all 4 clades of the phylogenetic tree (Fig. S2). Consequently, the Ascomycota CHSs (832 in total) (Fig. S2, Table S6) were used to construct a master tree to represent the major picture of the evolutionary history of the fungal CHSs. The resulting tree had 7 major clades with high bootstrap support (Fig. 1D), but the subclades were supported with low bootstrap value (<50%). The phylogeny of the 832 CHSs was consistent with their domain structure; in each major clade, the CHSs had the same domain structure. Therefore, further characterization of the evolution of the fungal CHSs could not be achieved based on phylogenetic and domain structure analyses. Unexpectedly, the 7 clades corresponded exactly to the 7 classes of CHSs that were previously reported based on a small number of fungal CHSs^5^,^10^,^11^,^12^,^13^. Consequently, we used the previously proposed nomenclature to name the 7 clades. Class I (designated CHSI below, and this naming system was also used for other classes of CHS), II and III belonged to Division 1, all of which contained the Type B3 domain structure (PF08407- PF01644-CON1). Division 2 contained CHSIV, CHSV and CHSVII. Both CHSV and CHSVII had a Type B4 domain structure (PF00063, PF00173, PF03142 and PF08766), and CHSIV had a Type B2 domain structure (PF00173 and PF03142). Division 3 contained only CHSVI with a Type B1 domain structure (PF03142).

**Figure 2:**
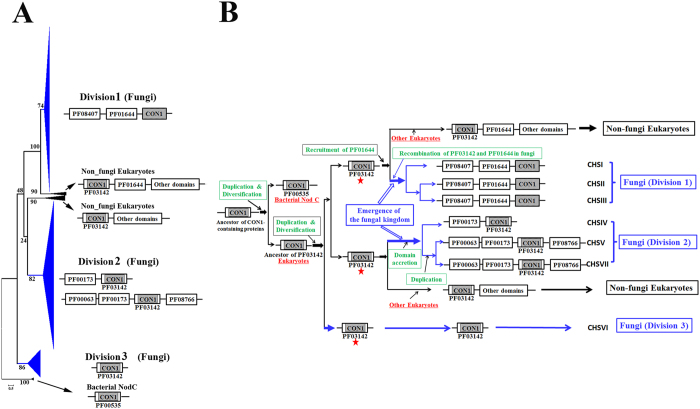
Analysis of the evolutionary origin of fungal CHSs. (**A**) The phylogenetic tree was constructed using the CON1 region from Ascomycota fungal CHSs (refer to Fig. 3D) and 82 CHSs from 22 non-fungi eukaryotes. The domain structure of proteins in each clade is shown on the right. Numbers at the nodes represent maximum-likelihood bootstrap values. (**B**) A deduced evolutionary trajectory of fungal CHSs based on gene duplication, domain recombination and accretion. Blue: evolution of the domain structure of fungal CHSs; green: evolutionary mechanisms. Red stars indicate the 3 ancestors of the fungal CHSs.

To investigate the evolutionary origin of fungal CHSs, we retrieved CHS sequences from non-fungi eukaryotes. Using fungal CHSs as queries, we retrieved all available (82 in total) CHSs in 22 species from Metazoa, Choanoflagellida, Amoebozoa, Alveolata and Stramenopiles, and their domain structure were also analyzed (Table S7). Ten bacterial NodC proteins were extracted from *Rhizobium*. spp (Table S8) and used as an outgroup for the phylogenetic analysis of eukaryotic CHSs. Their CON1 regions were extracted and included in the fungal CHSs (Table S6) for the phylogenetic analysis. The resulting tree contained 4 major clades (Fig. 2A). The clade containing bacterial NodCs was basal to eukaryotic CHSs. The Division 3 CHSs (CHSVI) themselves formed a major clade. The third major clade contained Division 1 CHSs and 45 non-fungi eukaryotic CHSs (from Alveolata or Stramenopiles) that had domains PF01644 and PF03142, and the fourth clade comprised Division 2 CHSs and 37 non-fungi eukaryotic CHSs (from Choanoflaggelida, Metazoa, Amoebozoa or Stramenopiles) that had domains PF03142 but not PF01644.

### CHS classification, and expansion and contraction of the CHS gene family

Because the master tree (Fig. 1D) contains 7 CHS classes determined by the domain structure and phylogenetic analysis, we used it (Fig. 1D) to characterize fungal CHSs in a species-by-species manner. To achieve this goal, the CON1 regions of CHSs from the 231 fungal species were included species by species into the dataset of the master tree (deleted, if any, repeat sequences) to construct new phylogenetic trees. Most CHSs (1, 305) were classified into the 7 CHS classes (Class I to VII) with high bootstrap support (>60%), and within each class, all CHSs had the same domain structure. Thirty-two CHSs, which had the same domain structure (PF08407-PF01644-CON1) as Division 1 CHSs, were basal with high bootstrap support (>60%) to the major clade containing CHSII and CHSIII in Division 1, and these were classified as CHS23b. The remaining 234 CHSs could not be grouped into any class based on phylogenetic and domain structure analysis, and thus designated as unclassified. Notably, 199 of the 234 unclassified CHSs, which had Type B1 domain structure, were those that were excluded in the phylogenetic analysis (Fig. S2), further demonstrating that they could have a complex evolutionary history. Therefore, alongside with domain structure analysis, the master tree (Fig. 1D) is suitable for classifying fungal CHSs. The classification of CHSs in all 231 fungal species is summarized in Table S2, and the number of CHSs by species is presented in Table S1.

**Figure 3:**
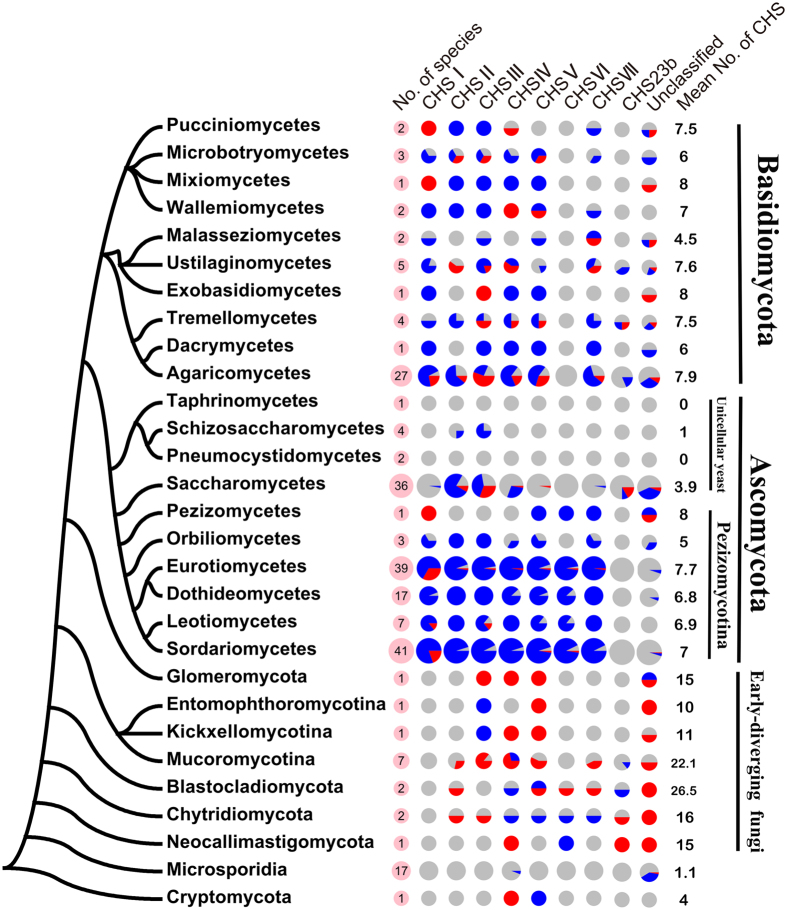
Distribution of the different classes of CHSs in the 9 fungal phyla. The left tree is the modified fungal life tree described in the Results section. Right pies show the distribution of different classes of CHSs in different fungi, and the number of species in a taxon is shown in its corresponding pink pie. Portions of a colored pie diagram show the percentage of the total number of species (in pink pies) in a taxon with multiple members (red), one member (blue), and no members (grey) within a CHS class. Mean No. of CHS: the average number of CHSs per species in a taxon.

To illustrate the expansion and contraction of the CHS gene family, the number of CHSs in each of the 231 fungal species was mapped to a fungal phylogenetic tree that was modified from the fungal life tree at JGI by replacing the parts consisting of Basidiomycota, Ascomycota, and Zygomycota with published phylogenetic trees^31^ (Fig. 3). The average number of CHSs in the Ascomycota varied greatly among the subphyla; for example, Pezizomycota had 6.9 CHSs per species, Saccharomycotina had 3.9 and Taphrinomycotina had 0.6. Only the Pezizomycota fungi carried all 7 classes of CHSs. No CHSs were identified in three Taphrinomycotina fungi. The average number of CHSs was relatively invariable in the Basidiomycota; the number ranged from 6 to 8 in 9 of 10 classes of the Basidiomycota fungi, and only the class Malasseziomycetes had 4.5 CHSs per species. No CHSVI (Division 3) was found in the Basidiomycota.

The average number of CHSs in the early-diverging fungi (in this study, refer to fungi from the Glomeromycota, Zygomycota, Blastocladiomycota, Chytridiomycota and Neocallimastigomycota) was 16.5. Strikingly, almost all of the early-diverging fungi had multiple unclassified CHSs that had a Type B1 domain structure. CHSVI was identified in only 3 early-diverging fungal species.

Only one microsporidial species (*Enterocytozoon bieneusi*) had 5 CHSs, and 2 species lacked CHSs. All of the other 14 species had 1 CHS. Excluding *Edhazardia aedis*, which had CHS IV, the other microsporidial species only had the unclassified CHSs with a Type B1 domain structure. In the basal fungus *Rozella allomycis* in Cryptomycota, only Division 2 CHSs (CHSIV and CHSV) were identified.

### CHSV and CHSVII produced by gene duplication contribute to heat stress tolerance in Pezizomycotina fungi

**Figure 4:**
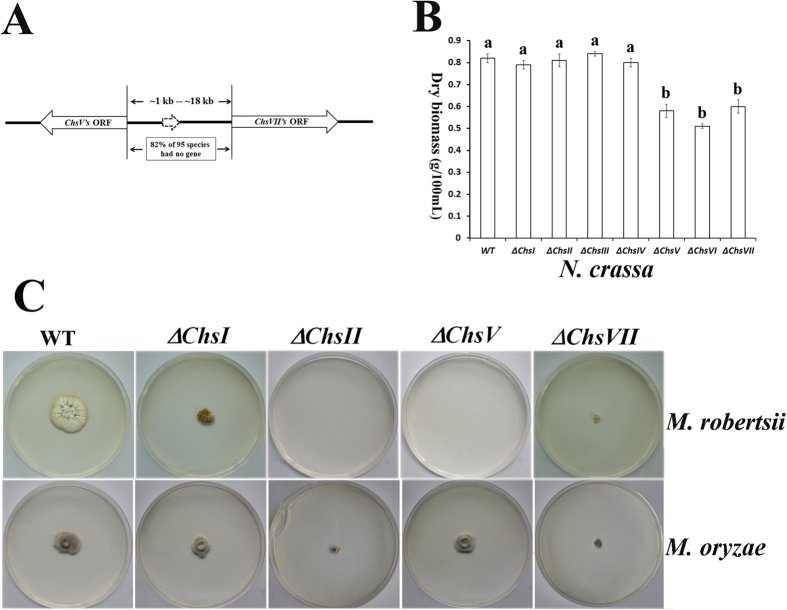
Heat stress tolerance of *Chs* mutants of *M. oryzae, N. crassa* and *M. robertsii*. (**A**) Orientation of *ChsV* and *ChsVII* on the chromosome of 95 Pezizomycotina fungi (Table S10). (**B**) Heat stress tolerance of *N. crassa*, as shown by the dry biomass grown in 100 mL of Sabouraud dextrose broth. Values with different letters are significantly different (*P* < 0.05, Kruskal-Wallis test). (**C**) Heat stress tolerance of *M. robertsii* and *M. oryzae*, as shown by growth on PDA plates. WT: wild type strain; *ΔChsI:* a mutant carrying a disruption in *ChsI*. This naming system is also used for other *Chs* genes.

CHSV and CHSVII had the same domain structure, and they were simultaneously found in 22 Basidiomycota, 100 Ascomycota and 1 early-diverging fungal species (Table S9). In the Basidiomycota and early-diverging fungi, *ChsV* and *ChsVII* were located randomly in the genomes. In contrast, *ChsV* and *ChsVII* exhibited a head-to-head arrangement on the chromosome in 95 species from Pezizomycotina Ascomycota (Fig. 4A, Table S10). In addition, all of these genes had introns, suggesting that *ChsV* and *ChsVII* were generated by tandem replication. Seventeen of the 95 Ascomycota fungi had a predicted gene encoding a hypothetical protein between the two CHS genes, and 78 had no gene between them.

Among the 95 Pezizomycotina fungi with *ChsV* and *ChsVII*, 28 species are pathogenic fungi to mammals that should be thermotolerant, and several others have been reported to be thermotolerant or thermophilic, such as *Chaetomium thermophilum* (Table S10), or they can survive under heat stress, such as *M. robertsii, N. crassa* and *M. oryzae*^32^,^33^,^34^. In previous studies, *ChsV* and *ChsVII* in the mammal pathogenic fungus *A. fumigatus* have been found to be involved in tolerance to heat stress^35^,^36^. These data suggest that *ChsV* and *ChsVII* could be involved in heat stress tolerance. Thus, we thus evaluated the heat stress tolerance of *Chs* mutants in 3 other representative fungi: the plant pathogenic fungus *M. oryzae*, the saprophytic fungus *N. crassa*, and the insect pathogenic fungus *M. robertsii* (in this study). There is one gene for each of 7 CHS classes in the 3 fungi, and all *Chs* genes had been previously disrupted in *M. oryzae*^12^,^37^; however, their relationship with heat stress tolerance has not yet been studied. In the present study, we disrupted all 7 *Chs* genes in *M. robertsii* (Table S11). Details of the gene disruption and subsequent complementation are described in Fig. S3. All assays showed that none of the 7 complemented *Chs* mutant strains were significantly different from the wild type strain (WT), and consequently we did not describe these data and results in the text; however, they are presented in the figures and tables.

In *N. crassa*, growth of the *ChsV* and *ChsVII* mutants was significantly inhibited by heat stress; *ChsVI* was also involved in heat stress tolerance (Fig. 4B). Similarly, *ChsV* and *ChsVII* were also important for heat stress tolerance in *M. robertsii*; growth of the *ChsVII* mutant was severely inhibited, and the *ChsV* mutant cannot grow under heat stress. In addition, *ChsI* and *ChsII* were also related to heat stress tolerance in *M. robertsii* (Figs 4C and S4A,B and C, Table S12). In *M. oryzae*, growth of the *ChsVII* mutant was inhibited in response to heat stress, but disruption of *ChsV* had no impact on heat stress tolerance. In addition, *ChsII* was also needed for heat stress tolerance in *M. oryzae* (Figs 4C and S4A and D, Table S12).

### CHSV and CHSVII contribute to pathogenicity in *M. robertsii*

**Figure 5:**
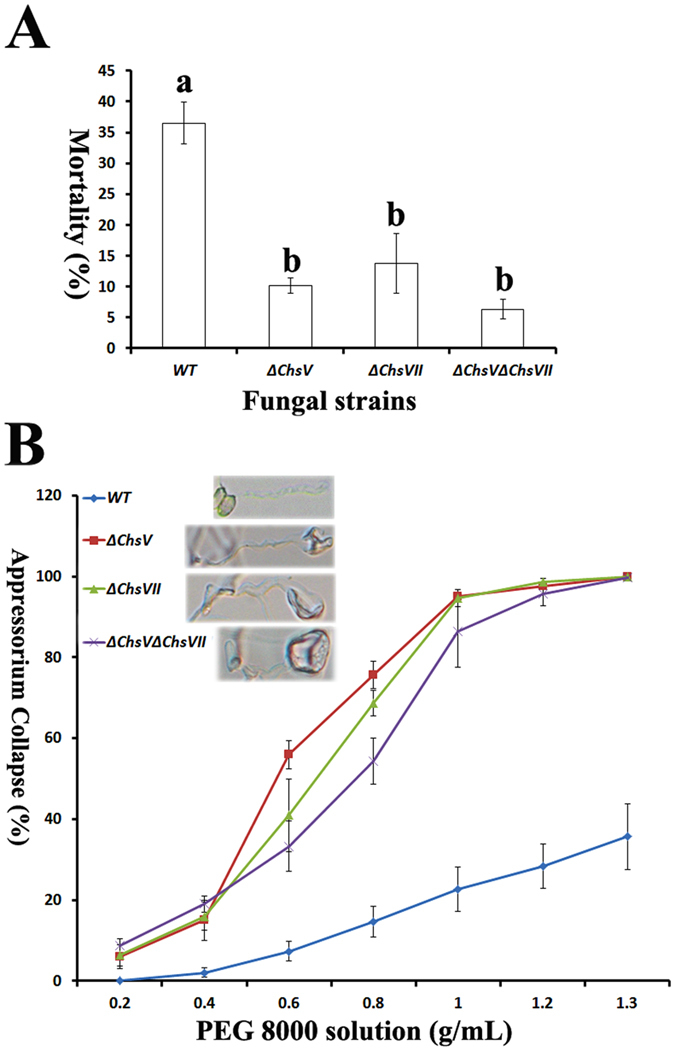
Pathogenicity of *M. robertsii Chs* mutants and WT. Mortality caused by *Chs* mutants and WT at 18 days post-inoculation. Insects were inoculated via a topical application of 1 × 10^6^ conidia/mL. Values with different letters are significantly different (*P* < 0.05, Kruskal-Wallis test). (**B**) Percentage of appressoria that collapsed following immersion in serial solutions of PEG-8000 (PEG) for 10 min. The image on the right of each label shows the collapsed appressoria.

*Chs* genes are involved in pathogenicity in mammal pathogenic fungi and many plant pathogenic fungi^12^,^13^,^18^,^25^,^33^,^34^, but their functions in pathogenicity have not been studied in insect pathogenic fungi. In the present study, we investigated the functions of *Chs* genes in the model insect pathogenic fungus *M. robertsii*. Compared with WT *M. robertsii*, only *ΔChs V* and *ΔChs VII* exhibited reduced virulence. The mortality caused by WT was 45%, which was significantly higher than that of *ΔChsV* and *ΔChsVII* (~15% mortality) (Fig. 5A, Table S13). The pathogenicity of the 2 mutants was not significantly different from each other. Double deletion of *ChsV* and *ChsVII* did not further decrease virulence (Fig. 5A).

Compared with WT, *ΔChsV, ΔChsVII* and *ΔChsVΔChsVII* produced deformed appressoria (the infection structure) containing a large vacuole-like structure (Fig. S5). Analysis of appressorial collapse in PEG-8000 solutions revealed that the loss of *ChsV* and *ChsVII* resulted in a dramatic reduction of turgor pressure (Fig. 5B), and double deletion of *ChsV* and *ChsVII* did not further reduce the appressorial turgor pressure.

### CHSV and CHSVII contribute to morphogenesis in *M. robertsii*

**Figure 6:**
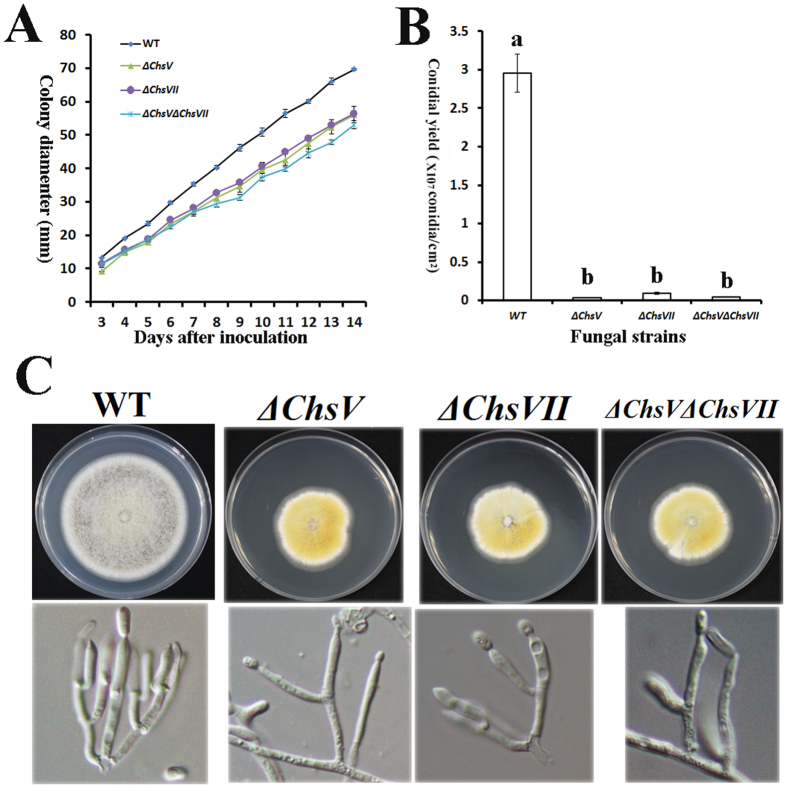
Morphological analysis of *M. robertsii Chs* mutants and WT. (**A**) Growth curve of WT and *Chs* mutants on PDA plates. (**B**) Conidial yields (mean ± SE) of WT and *Chs* mutants on PDA plates. Conidial yields were determined at 15 days post-inoculation. Values with different letters are significantly different (*P* < 0.05, Kruskal-Wallis test). (**C**) Colony morphology (upper panel) and conidiophores (lower panel) of WT and *Chs* mutants.

We also tested the involvement of *Chs* genes in saprophytic growth in *M. robertsii*. On potato dextrose agar plates (PDA), the growth and conidial yields of *ΔChsII, ΔChsIII, ΔChsIV* and *ΔChsVI* were not significantly different from those of WT; however, compared with WT, the growth of *ΔChsI, ΔChsV* and *ΔChsVII* was significantly inhibited (Fig. 6A, Table S14). *ΔChsV* and *ΔChsVII* produced yellow pigments with hyphae that were firmly attached to the agar (Figs 6C and S6).

*ΔChsV, ΔChsVII* and *ΔChsVΔChsVII* had impaired conidiophores (conidium-producing structure) (Figs 6C and S6), and thus they had a significantly lower conidial yield than WT (Fig. 6B). There were no significant differences in conidial yield among the 3 mutants (Fig. 6B, Table S15).

## Discussion

In the present study, two major mechanisms were found to drive the evolution of the CHS gene family, resulting in at least 7 classes of CHSs in the fungal kingdom. One mechanism is gene duplication and subfunctionalization. The second mechanism is domain recombination and domain accretion, which occurred in the paralogs generated from gene duplications, as supported by the consistency between the CHS phylogeny and domain structure observed herein (Fig. 2B). Fungal CHSs could have 3 different ancestors that potentially carry domain PF03142, which could share an ancestor with bacterial NodC proteins (Fig. 2B). Rhizobia might acquire NodCs via horizontal gene transfer^38^, but our phylogenetic analyses showed that rhizobial NodCs reside within a clade that is separated from eukaryotic CHSs. CHSVI proteins could have their own distinct ancestor, and currently existing CHSVI proteins could retain this ancestral function. According to the consensus conducted in the present study, CHSVI could be specific to fungi; however, this hypothesis remains to be confirmed by a more exhaustive search of CHSs from non-fungi eukaryotes. The ancestor of Division 1 CHSs could be shared by CHSs from the kingdoms Alveolata and Stramenopiles. Fungal Division 1 CHSs contained the CON1 region (also found within domain PF03142) following domain PF01644. The non-fungi eukaryotic CHSs that share an ancestor with fungal Division 1 CHSs contained intact PF03142 and PF01644. Thus, it is likely that the ancestor of Division 1 CHSs had intact PF03142 and PF01644, but recombination led to the loss of the N-terminus of PF03142, resulting in region PF01644-CON1 (Figs 1B and 2B), which is consistent with a previously proposed hypothesis^6^,^10^. This recombination could have occurred during the early stage of the emergence of the fungal kingdom because Division 1 CHSs are present in Ascomycota, Basidiomycota and early-diverging fungal phyla. The product of the domain recombination duplicated twice, resulting in CHSI, CHSII and CHSIII.

Fungal Division 2 CHSs have an intact PF03142, and they could share an ancestor with CHSs from the kingdoms Choanoflaggelida, Metazoa, Amoebozoa or Stramenopiles. The ancestor of Division 2 CHSs could experience domain accretion (Fig. 2B), resulting in CHSIV, CHSV and CHSVII; this ancestor could first recruit PF00173 because all CHSIV, CHSV and CHSVII proteins have PF00173, and the CHSIV was basal to CHSV and CHSVII in the phylogenetic tree (Fig. 1D). The recruitment of PF00173 could occur during the emergence of the fungal kingdom because we identified only one PF00173-containing CHS (XP_002291658) from a non-fungi eukaryote (*Thalassiosira pseudonana* CCMP1335). *T. pseudonana* could have acquired PF00173 via independent domain recruitment. In the fungal kingdom, the protein resulting from the recruitment of PF00173 could duplicate, with one paralog retaining its original domain structure to become CHSIV. The other paralog could have undergone domain accretion by recruiting PF00063 and PF08766, and the resulting gene duplicated to produce CHSV and CHSVII. Although PF00063 was also identified in 4 CHSs in lophotrochozoans, fungal and lophotrochozoan PF00063 emerged from different kinds of myosin^39^. Therefore, the recruitment of PF00063 and PF08766 by fungal CHSs could only have occurred in the fungal kingdom, potentially during the early stage of emergence of the kingdom because both dikarya fungi and early-diverging fungi contain CHSs with PF00063 and PF08766. Because the basal fungus *Cryptomycota* contains CHSIV and CHSV, but not CHSVII, the ancestor of CHSV and CHSVII might have undergone a duplication event after the divergence of the ancestor of the basal fungus from that of other fungi; this duplication could have occurred independently in taxonomically distinct fungi. In the Basidiomycota and early-diverging fungi, this duplication event could be ectopic because *ChsV* and *ChsVII* appeared to be scattered randomly throughout the genomes; in contrast, in the Ascomycota, a tandem duplication could have occurred because *ChsV* and *ChsVII* exhibit a head-to-head arrangement on the chromosome in 95% of this group of fungi. This tandem duplication could have taken place during the emergence of the Pezizomycota, but it is also possible that it occurred during the emergence of the Ascomycota with a subsequent loss of the product in subphyla other than the Pezizomycota.

The emergence of CHSV and CHSVII is important for the development of fungal heat stress tolerance and pathogenicity in pathogenic fungi. Heat stress is a common abiotic stress that many fungi encounter in nature. In this study, we found that CHSV and CHSVII are involved in heat stress tolerance in 3 Pezizomycotina fungi: *M. robertsii, M. oryzae* and *N. crassa*. Previous studies have shown that CHSV and CHSVII are also involved in heat stress tolerance in *A. fumigatus*^35^,^36^. It is not surprising that more fungi will be found to use CHSV and CHSVII for tolerance to heat stress. Tolerance to heat stress provided by CHSV and CHSVII are important for pathogenicity in some pathogenic fungi. As a mammal pathogenic fungus, *A. fumigatus* must overcome the host body temperature to achieve successful infection. Fungal infection induces behavioral fever in some insects such as locusts, in which insects raise their body temperature as a means of literally toasting a fungal invader; CHSV and CHSVII could be required by *M. robertsii* to evade such behavioral immunity. CHSV and CHSVII are also involved in the formation of the infection structure (appressorium) in *M. robertsii*. As in *M. robertsii*, CHSV and CHSVII are also involved in appressorial formation in plant pathogenic fungi^12^,^16^,^17^,^18^,^23^. In addition to heat stress tolerance and the development of the infection structure, CHSV and CHSVII are involved in hyphal growth and it is axiomatic that they are important for *M. robertsii* to proliferate in insects and thus for infection. Therefore, the emergence of CHSV and CHSVII is important for many fungi to adapt to their respective ecological niches.

Diversities also exist in the biological functions of CHSV and CHSVII among different fungi. For example, both CHSV and CHSVII are important for heat stress tolerance in *M. robertsii, N. crassa* and *A. fumigatus*, but only CHSVII (not CHSV) is involved in heat stress tolerance in *M. oryzae*. In addition, CHSV and CHSVII are independently, i.e., not redundantly and compensatorily, important for aerial hyphal growth and conidiation in *M. robertsii* (in this study). In contrast, CHSV and CHSVII in *A. nidulans* perform compensatory functions in hyphal growth and conidiation^20^,^21^,^22^. The diversified biological functions of CHSV and CHSVII could be a consequence of diverse subfunctionalization processes, which might have occurred in different fungi after the duplication resulting in CHSV and CHSVII. In addition, evolutionary changes in the regulatory region, timing or location of the gene expression of CHSV and CHSVII could also be attributed to the diverse biological functions of these two CHSs among different fungi.

Based on the above analysis, all classes of fungal CHSs resulted from ancient domain recombination, domain accretion and gene duplications. However, the expansion and loss of CHSs have been commonly observed in the fungal kingdom. The loss of CHSs appears to be morphology-specific because it occurred most frequently in unicellular fungi (Fig. 3). Division 2 and 3 CHSs were lost in the yeast fungi. A significant loss of CHSs also occurred in the unicellular Microsporidia. Moreover, the evolution of CHSs in Microsporidia appears to differe from that in other fungi; excluding *Edhazardia aedis*, which has CHS IV, 14 other microsporidial species only contain the unclassified CHSs containing the Type B1 domain structure. Microsporidia have the smallest known (nuclear) eukaryotic genomes because their parasitic lifestyle has led to the loss of many genes^40^. The retention of CHSs in most Microsporidia suggests that chitin is a key component for this group of fungi to maintain their parasitic lifecycle.

The expansion of CHSs appears to be lineage-specific. Clear expansion has been observed in all 15 early-diverging fungi, with 16.5 CHSs per species, which is almost 3 times greater than the 5.8 CHSs per species determined in dikarya fungi (Fig. 3). A clear characteristic of the CHS family in early-diverging fungi is that they all contain multiple copies of the unclassified CHS that has a Type B1 domain structure, with *Allomyces macrogynus* having the largest copy number (16) of such a CHS. The expansion of CHSIII and CHSIV has also been observed in most early-diverging fungi. Compared with the Ascomycota and Basidiomycota fungi, little is known about the biology of the early-diverging fungi and the biological functions of CHSs. Therefore, it is currently difficult to determine the contribution of the expansion of CHSs to the environmental adaptation of early-diverging fungi.

In conclusion, we identified the full complement of CHSs from 231 fungal species encompassing 9 phyla. Using the largest dataset to date, we characterized the evolution of the CHS gene family in the fungal kingdom based on phylogenetic and domain structure analyses. Gene duplication, domain recombination and accretion are major mechanisms underlying the diversification of the fungal CHS gene family. Contraction of the CHS gene family appears to be morphology-specific, with a significant loss of CHSs in unicellular fungi; in contrast, expansion is lineage-specific, with obvious expansion in early-diverging fungi. CHSV and CHSVII that have the same domain structure were produced by recruitment of the domains PF00063 and PF08766 and subsequent duplications; a comparative analysis of their biological functions in different fungi showed that such evolutionary events are important for the morphogenesis of filamentous fungi, development of pathogenicity in pathogenic fungi, and heat stress tolerance in Pezizomycotina fungi. The present work significantly advances our understanding of the evolution of the CHS gene family in the fungal kingdom and its correlation with fungal morphogenesis and adaptation to ecological niches.

## Materials and Methods

### Identification of CHSs by HMM

An HMM search was conducted to identify the full complement of CHS family members from a fungal genome. The protein databases of 231 fungal species were individually downloaded from NCBI (National Center for Biotechnology) or JGI (Joint Genome Institute, with the consent of the project’s principal investigators). Twenty-four CHSs that have been functionally characterized in previous studies (Table S16) were used as queries for training to determine the HMM parameters for searching the fungal genomes for CHSs. We used MUSCLE (default parameters) to align the reference proteins, and the commands *hmmbuild* and *hmmsearch* in HMMER 3.1b1 (http://hmmer.org/) to implement the search experiments with default parameters (E-value = 10).

Three selection criteria were used to remove false positives from the HMM search. The first criterion was the presence of characteristic CHS domains (cd04190 from the CDD database and PF03142 from the Pfam database). The second criterion was the presence of conserved CON1 motifs (QxxEY, EDRxL, and QxRRW)^5^,^6^. After the above 2 rounds of removal, repeated sequences were manually deleted.

The CHSs from non-fungi eukaryotes and the NodC proteins from *Rhizobium* were manually downloaded from the NCBI databases.

### Phylogeny and domain structure analysis

The CON1 region in all CHSs was extracted using a local Perl script for the phylogenetic analysis. The protein sequences were aligned using MUSCLE 3.7 with default parameters^29^. The alignments were then manually refined and end-trimmed to eliminate poor alignments and divergent regions. Unambiguously aligned positions were used to construct ML trees with MEGA version 6.0 (gap treatment: partial deletion; model of evolution: WAG model; 100 bootstrap replications)^41^.

Domains in CHSs were identified using the Batch Search program provided in the PFAM database (http://pfam.xfam.org/).

### Gene disruption in *M. robertsii*

*M. robertsii* ARSEF2575 was cultured as previously described^41^. *Escherichia coli* DH5α was used for plasmid construction. *Agrobacterium tumefaciens* AGL1 was used for fungal transformation as previously described^42^.

Gene disruption based on homologous recombination was conducted according to our previously developed high-throughput gene disruption methodology^43^. The details of all primers used in this study are provided in Table S17. To complement a gene disruption mutant, the genomic clone of the gene containing the promoter region, ORF (open reading frame) and termination region was amplified using high-fidelity Taq DNA polymerase (KOD, Japan) and inserted into pFBENGFP to construct the gene complementation plasmid^42^.

### Conidiation assays

Conidial yields were determined after 18 days of incubation as previously described^44^. Conidiophores were observed daily beginning at 3 days of incubation as previously described^44^.

### Pathogenicity assays

Development of the appressorium was observed against the hindwing of *L. migratoria manilensis* and on the hydrophobic surface of the Petri dish (Corning, USA) as previously described^45^. Appressorial turgor pressure was tested as previously described^46^. PEG8000 solutions with concentrations ranging from 10% to 140% (w/v) were used to generate turgor pressure. Appressoria were formed in 0.01% YE (yeast extract) on the hydrophobic surface of a plastic Petri dish with a diameter of 3 cm (Corning USA). The medium was poured off, and a PEG solution (2 mL) was added and incubated for 10 min. The percentage of collapsed appressoria was determined for 200 cells per PEG solution.

Bioassays were conducted using last instar *G. mellonella* larvae (Ruiqingbait Co., Shanghai, China) as previously described^45^. Inoculations by topical application were performed by immersing the insects in the conidial suspensions. Mortality was recorded daily, and the LT_50_ (the time required to kill 50% of the *G. mellonella* larvae) was determined using the *SPSS* Statistical Package (SSPS Inc., Chicago, IL). All bioassays were repeated three times with 30 insects per replicate. *ΔChsV*, Δ*ChsVII*, and *ΔChsVΔChsVII* had a very low conidial yield, and sufficient conidia could not be obtained to prepare a conidial suspension at 10^7^ conidia/mL, which is a common concentration used for bioassays of *M. robertsii*^45^. Conidial suspensions at 10^6^conidia/mL were thus used in this study.

### Heat stress tolerance assay

Tolerance to heat stress was tested by measuring growth on PDA at 35 °C for *M. robertsii* and *M. oryzae*. For *M. robertsii*, 5 μL of the conidial suspension (10^5^conidia/mL) was applied to the center of a PDA plate and then allowed to grow. For *M. oryzae*, 200 μL of the conidial suspension (10^4^ conidia/mL) was evenly plated on a PDA plate and allowed to grow at 27 °C for 5 days; a piece of mycelium with agar (diameter = 3 mm) was obtained from the plate and placed at the center of a new PDA plate and allowed to grow. The diameter of the colony was recorded daily for 5 days after inoculation. *N. crassa* produces fluffy a colony on PDA plates, and its colony diameter cannot be measured; its growth under heat stress was thus characterized by measuring the dry biomass cultured in Sabouraud dextrose broth (10^4^ conidia in 100 mL of medium) at 42 °C for 5 days.

## Additional Information

**How to cite this article**: Liu, R. *et al*. Evolution of the chitin synthase gene family correlates with fungal morphogenesis and adaption to ecological niches. *Sci. Rep.* **7**, 44527; doi: 10.1038/srep44527 (2017).

**Publisher's note:** Springer Nature remains neutral with regard to jurisdictional claims in published maps and institutional affiliations.

## Supplementary Material

Supplementary Figures

Supplementary Table S1

Supplementary Table S2

Supplementary Table S3

Supplementary Table S4

Supplementary Table S5

Supplementary Table S6

Supplementary Table S7

Supplementary Table S8

Supplementary Table S9

Supplementary Table S10

Supplementary Table S11

Supplementary Table S12

Supplementary Table S13

Supplementary Table S14

Supplementary Table S15

Supplementary Table S16

Supplementary Table S17

## References

[b1] Hawksworth D. L. The magnitude of fungal diversity: the 1.5 million species estimate revisited. Mycol Res 105, 1422–1432 (2001).

[b2] Gooday G. W. Physiology of microbial degradation of chitin and chitosan. In: Ratledge C., editor Biochemistry of Microbial Degradation. Dordrecht: Springer; p. 279–312 (1994).

[b3] Cid V. J. . Molecular basis of cell integrity and morphogenesis in *Saccharomyces cerevisiae*. Microbiol Rev 59, 345–386 (1995). 7565410 10.1128/mr.59.3.345-386.1995 PMC239365

[b4] Glaser L. & Brown D. H. The synthesis of chitin in cell-free extracts of Neurospora crassa. J Biol Chem 228, 729–742 (1957). 13475355

[b5] Choquer M., Boccara M., Gonc¸alves I. R., Soulie M. & Vidal-Cros A. Survey of the Botrytis cinerea chitin synthase multigenic family through the analysis of six euascomycetes genomes. Eur J Biochem 271, 2153–2164 (2004). 15153106 10.1111/j.1432-1033.2004.04135.x

[b6] Pacheco-Arjona J. R. & Ramirez-Prado J. H. Large-scale phylogenetic classification of fungal chitin synthases and identification of a putative cell-wall metabolism gene cluster in Aspergillus genomes. PLoS One 9, e104920 (2014). 25148134 10.1371/journal.pone.0104920 PMC4141765

[b7] Dorfmueller H. C., Ferenbach A. T., Borodkin V. S. & van Aalten D. M. A structural and biochemical model of processive chitin synthesis. J Biol Chem 289, 23020–23028 (2014). 24942743 10.1074/jbc.M114.563353 PMC4132801

[b8] Semino C. E. & Robbins P. W. Synthesis of NOD-like chitin oligosaccharides by the Xenopus developmental protein DG42. Proc Natl Acad Sci USA 92, 3498–3501 (1995). 7724589 10.1073/pnas.92.8.3498 PMC42194

[b9] Ruiz-Herrera J. & Ortiz-Castellanos L. Analysis of the phylogenetic relationships and evolution of the cell walls from yeasts and fungi. FEMS Yeast Res 10, 225–243 (2010). 19891730 10.1111/j.1567-1364.2009.00589.x

[b10] Mandel M. A., Galgiani J. N., Kroken S. & Orbach M. J. Coccidioides posadasii contains single chitin synthase genes corresponding to classes I to VII. Fungal Genet Biol 43, 775–788 (2006). 16857399 10.1016/j.fgb.2006.05.005

[b11] Riquelme M. & Bartnicki-Garcia S. Advances in understanding hyphal morphogenesis: Ontogeny, phylogeny and cellular localization of chitin synthases. Fungal Biol Rev 22, 56–70 (2008).

[b12] Kong L. A. . Different chitin synthase genes are required for various developmental and plant infection processes in the rice blast fungus Magnaporthe oryzae. PLoS Pathog. 8, e1002526 (2012). 22346755 10.1371/journal.ppat.1002526 PMC3276572

[b13] Gandía M., Harries E. & Marcos J. F. The myosin motor domain-containing chitin synthase PdChsVII is required for development, cell wall integrity and virulence in the citrus postharvest pathogen Penicillium digitatum. Fungal Genet Biol 67, 58–70 (2014). 24727399 10.1016/j.fgb.2014.04.002

[b14] Li M. . Evolution and functional insights of different ancestral orthologous clades of chitin synthase genes in the fungal tree of life. Front. Plant Sci. 7, 37 (2016). 26870058 10.3389/fpls.2016.00037 PMC4734345

[b15] Liu J. J., Sturrock R. & Ekramoddoullah A. K. The superfamily of thaumatin-like proteins, its origin, evolution, and expression towards biological function. Plant Cell Rep 29, 419–436 (2010). 20204373 10.1007/s00299-010-0826-8

[b16] Amnuaykanjanasin A. & Epstein L. A class V chitin synthase gene, chsA is essential for conidial and hyphal wall strength in the fungus *Colletotrichum graminicola (Glomerella graminicola*). Fungal Genet Biol 38, 272–285 (2003). 12684017 10.1016/s1087-1845(02)00563-7

[b17] Amnuaykanjanasin A. & Epstein L. A class Vb chitin synthase in *Colletotrichum graminicola* is localized in the growing tips of multiple cell types, in nascent septa, and during septum conversion to an end wall after hyphal breakage. Protoplasma 227, 155–164 (2006). 16520880 10.1007/s00709-005-0126-2

[b18] Larson T. M., Kendra D. F., Busman M. & Brown D. W. Fusarium verticillioides chitin synthases CHS5 and CHS7 are required for normal growth and pathogenicity. Curr Genet 57, 177–189 (2011). 21246198 10.1007/s00294-011-0334-6

[b19] Mellado E., Dubreucq G., Mol P., Sarfati J., Paris S., Diaquin M. . Cell wall biogenesis in a double chitin synthase mutant (chsG-/chsE-) of Aspergillus fumigatus. Fungal Genet Biol 38, 98–109 (2003). 12553940 10.1016/s1087-1845(02)00516-9

[b20] Takeshita N., Ohta A. & Horiuchi H. CsmA, a gene encoding a class V chitin synthase with a myosin motor-like domain of Aspergillus nidulans, is translated as a single polypeptide and regulated in response to osmotic conditions. Biochem Biophys Res Commun 298, 103–109 (2002). 12379226 10.1016/s0006-291x(02)02418-x

[b21] Takeshita N., Ohta A. & Horiuchi H. CsmA, a class V chitin synthase with a myosin motor-like domain, is localized through direct interaction with the actin cytoskeleton in Aspergillus nidulans. Mol Biol Cell 16, 1961–1970 (2005). 15703213 10.1091/mbc.E04-09-0761 PMC1073675

[b22] Takeshita N., Yamashita S., Ohta A. & Horiuchi H. Aspergillus nidulans class V and VI chitin synthases CsmA and CsmB, each with a myosin motor-like domain, perform compensatory functions that are essential for hyphal tip growth. Mol Microbiol 59, 1380–1394 (2006). 16468983 10.1111/j.1365-2958.2006.05030.x

[b23] Weber I., Assmann D., Thines E. & Steinberg G. Polar localizing class V myosin chitin synthases are essential during early plant infection in the plant pathogenic fungus Ustilago maydis. Plant Cell 18, 225–242 (2006). 16314447 10.1105/tpc.105.037341 PMC1323495

[b24] Fajardo-Somera R. A., Jöhnk B., Bayram Ö., Valerius O., Braus G. H. & Riquelme M. Dissecting the function of the different chitin synthases in vegetative growth and sexual development in Neurospora crassa. Fungal Genet Biol 75, 30–45 (2015). 25596036 10.1016/j.fgb.2015.01.002

[b25] Soulie M. C. . Botrytis cinerea virulence is drastically reduced after disruption of chitin synthase class III gene (Bcchs3a). Cell Microbiol 8, 1310–1321 (2006). 16882034 10.1111/j.1462-5822.2006.00711.x

[b26] Wang Z. . WdChs2p, a class I chitin synthase, together with WdChs3p (class III) contributes to virulence in Wangiella (Exophiala) dermatitidis. Infect Immun 69, 7517–7526 (2001). 11705928 10.1128/IAI.69.12.7517-7526.2001 PMC98842

[b27] Motoyama T., Kojima N., Horiuchi H., Ohta A. & Takagi M. Isolation of a chitin synthase gene (chsC) of Aspergillus nidulans. Biosci Biotechnol Biochem 58, 2254–2257 (1994). 7765719 10.1271/bbb.58.2254

[b28] Rogg L. E., Fortwendel J. R., Juvvadi P. R., Lilley A. & Steinbach W. J. The chitin synthase genes chsA and chsC are not required for cell wall stress responses in the human pathogen Aspergillus fumigatus. Biochem Biophys Res Commun 411, 549–554 (2011). 21763289 10.1016/j.bbrc.2011.06.180 PMC3712863

[b29] Edgar R. C. MUSCLE: multiple sequence alignment with high accuracy and high throughput. Nucleic Acids Res 32, 1792–1797 (2004). 15034147 10.1093/nar/gkh340 PMC390337

[b30] Chothia C., Gough J., Vogel C. & Teichmann S. A. Evolution of the protein repertoire. Science 300, 1701–1703 (2003). 12805536 10.1126/science.1085371

[b31] James T. Y., Kauff F., Schoch C. L., Matheny P. B., Hofstetter V., Cox C. J. . Reconstructing the early evolution of Fungi using a six-gene phylogeny. Nature 443, 818–822 (2006). 17051209 10.1038/nature05110

[b32] Liao X., Lu H. L., Fang W. & St Leger R. J. Overexpression of a Metarhizium robertsii HSP25 gene increases thermotolerance and survival in soil. Appl Microbiol Biotechnol 98, 777–783 (2014). 24265026 10.1007/s00253-013-5360-5

[b33] Kapoor M., Sreenivasan G. M., Goel N. & Lewis J. Development of thermotolerance in Neurospora crassa by heat shock and other stresses eliciting peroxidase induction. J Bacteriol 172, 2798–2801 (1990). 2139653 10.1128/jb.172.5.2798-2801.1990 PMC208932

[b34] Li G. . MoSfl1 is important for virulence and heat tolerance in Magnaporthe oryzae. PLoS One. 6, e19951 (2011). 21625508 10.1371/journal.pone.0019951 PMC3098271

[b35] Muszkieta L. . Deciphering the role of the chitin synthase families 1 and 2 in the *in vivo* and *in vitro* growth of Aspergillus fumigatus by multiple gene targeting deletion. Cell Microbiol 16, 1784–1805 (2014). 24946720 10.1111/cmi.12326

[b36] Jiménez-Ortigosa C. . Chitin synthases with a myosin motor-like domain control the resistance of Aspergillus fumigatus to echinocandins. Antimicrob Agents Chemother 56, 6121–131 (2012). 22964252 10.1128/AAC.00752-12 PMC3497188

[b37] Mccluskey A. The Fungal Genetics Stock Center: From Molds to Molecules. Adv Appl Microbiol 52, 245–262 (2003). 12964247 10.1016/s0065-2164(03)01010-4

[b38] Bontemps C. . Burkholderia species are ancient symbionts of legumes. Mol Ecol. 19, 44–52 (2010). 20002602 10.1111/j.1365-294X.2009.04458.x

[b39] Zakrzewski A. C. . Early divergence, broad distribution, and high diversity of animal chitin synthases. Genome Biol Evol 6, 316–325 (2014). 24443419 10.1093/gbe/evu011 PMC3942024

[b40] Keeling P. J. & Fast N. M. MICROSPORIDIA: Biology and Evolution of Highly Reduced Intracellular Parasites. Annu Rev Microbiol 56, 93–116 (2002). 12142484 10.1146/annurev.micro.56.012302.160854

[b41] Tamura K., Stecher G., Peterson D., Filipski A. & Kumar S. MEGA6: Molecular Evolutionary Genetics Analysis version 6.0. Mol Biol Evol 30, 2725–2729 (2013). 24132122 10.1093/molbev/mst197 PMC3840312

[b42] Fang W., Pei Y. & Bidochka M. J. Transformation of *Metarhizium anisopliae* mediated by *Agrobacterium tumefaciens*. Can J Microbiol 52, 623–626 (2006). 16917517 10.1139/w06-014

[b43] Xu C., Zhang X., Qian Y., Chen X. X., Liu R., Zeng G. . A High-Throughput Gene Disruption Methodology for the Entomopathogenic Fungus *Metarhizium robertsii*. PLoS One 9, e107657 (2014). 25222118 10.1371/journal.pone.0107657 PMC4164657

[b44] Chen X. . MAPK cascade-mediated regulation of pathogenicity, conidiation and tolerance to abiotic stresses in the entomopathogenic fungus *Metarhizium robertsii*. Environ Microbiol 18, 1048–1062 (2016). 26714892 10.1111/1462-2920.13198

[b45] Zhao H. . Host-to-pathogen gene transfer facilitated infection of insects by a pathogenic fungus. PLoS Pathog. 10, e1004009 (2014). 24722668 10.1371/journal.ppat.1004009 PMC3983072

[b46] Fang W., Pava-ripoll M., Wang S. & St Leger R. J. Protein kinase A regulates production of virulence determinants by the entomopathogenic fungus, Metarhizium anisopliae. Fungal Genet Biol 46, 277–285 (2009). 19124083 10.1016/j.fgb.2008.12.001

