# Supplementary material for: Evolution of the chitin synthase gene family correlates with fungal morphogenesis and adaption to ecological niches
Source: Sci Rep. 2017 Mar 16;7:44527. doi: 10.1038/srep44527 (PMC5353729; doi:10.1038/srep44527)
Supplement: Supplementary Figures [file srep44527-s1.doc]

**Supplementary Figures**

**Title**: Evolution of the chitin synthase gene family correlates with fungal morphogenesis and adaption to ecological niches

Ran Liu^ξ1^ (liuran1990@hotmail.com), Chuan Xu^ξ1^ (bioxc@zju.edu.cn), Qiangqiang Zhang^1^ (21407022@zju.edu.cn), Shiyi Wang^1^ (3140102621@zju.edu.cn), Weiguo Fang^*,1,2^ ([wfang1@zju.edu.cn](mailto:wfang1@zju.edu.cn))

1. Institute of Microbiology, College of Life Sciences, Zhejiang University, Hangzhou, 310058, Zhejiang, China
2. Institute of Insect Sciences, Zhejiang University, Hangzhou 310058, Zhejiang, China

*Corresponding author: Weiguo Fang

Tel: 86-571-88206668

E-mail: wfang1@zju.edu.cn

ξ: The authors contribute equally to this paper

**
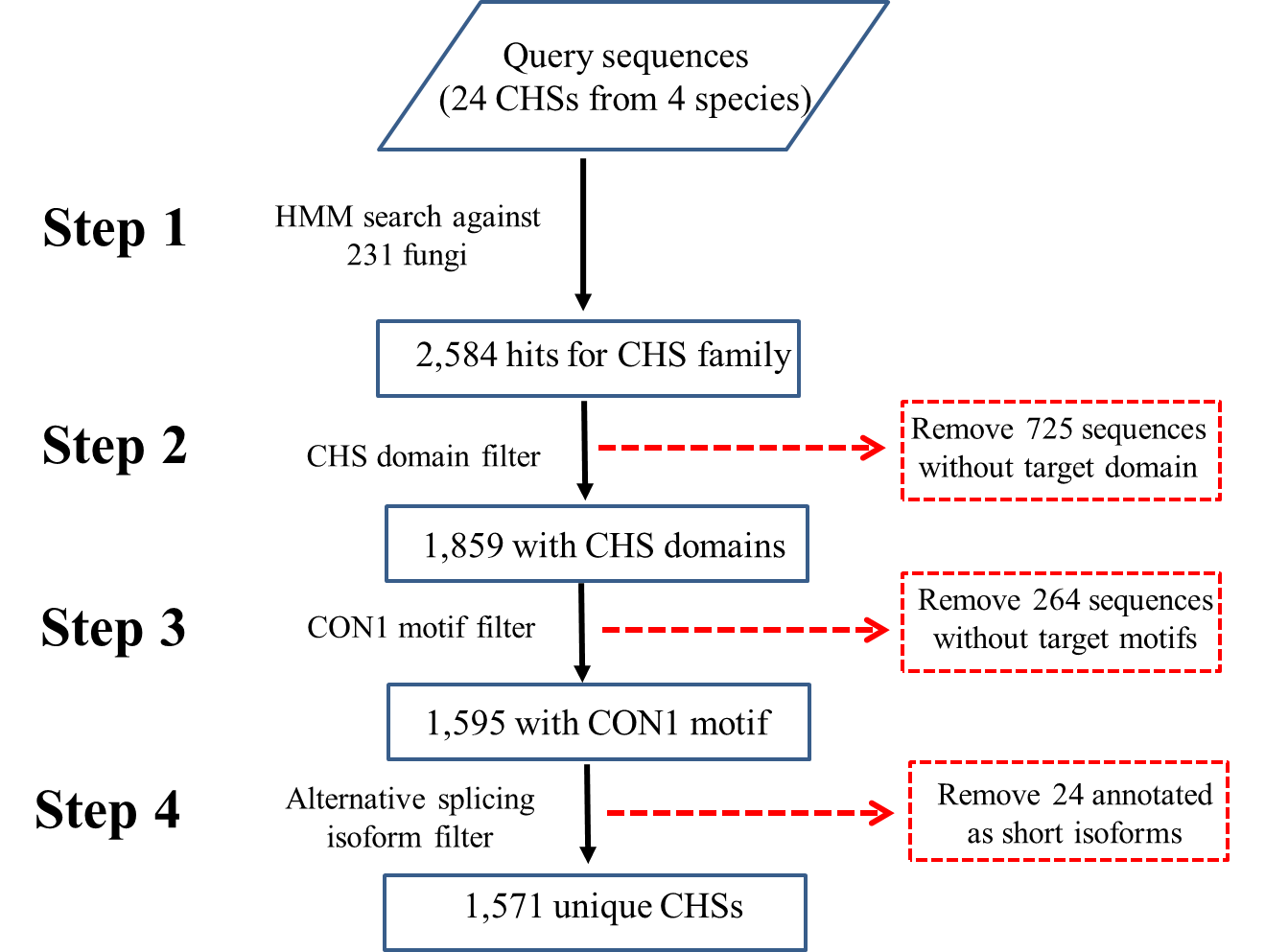
**

Fig. S1: Flow chart of the identification of CHSs from 231 fungal species

**
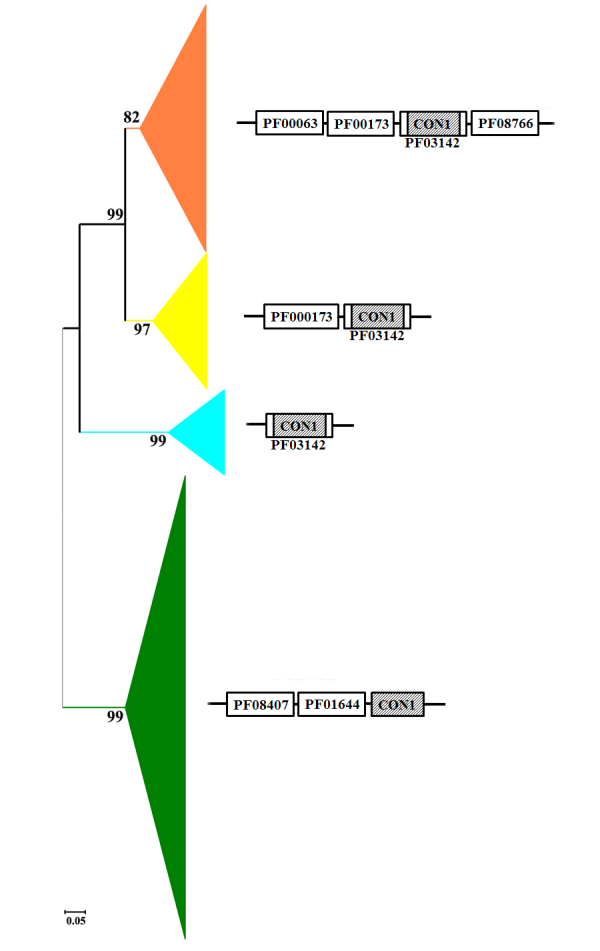
**

Fig. S2: Phylogeny and domain structures of 1, 283 CHSs. The phylogenetic tree was constructed using the protein sequence of the CON1 regions of fungal CHSs. The same color indicates proteins with the same domain structure indicated on the right. Numbers at nodes represent bootstrap values of maximum likelihood.

**
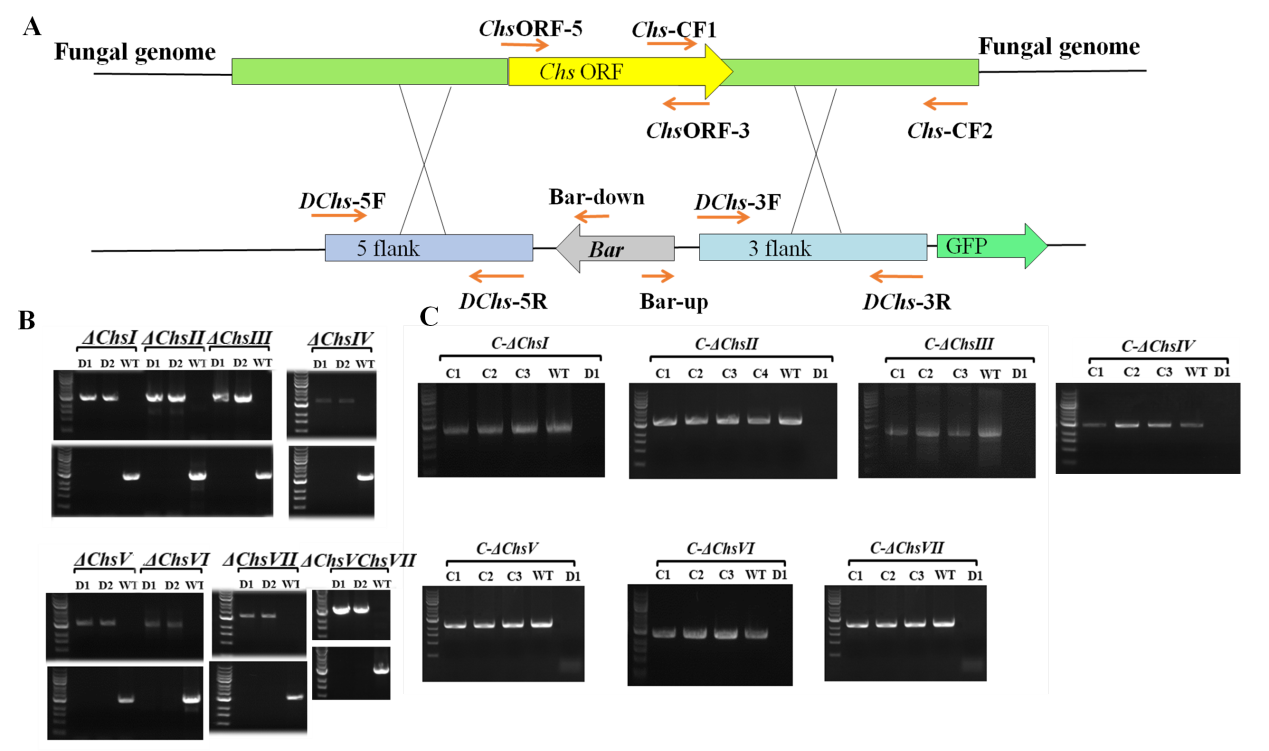
**

Fig. S3: Disruption of genes encoding the 7 CHSs in *M. robertsii*. (A) The disruption plasmid of a gene (bottom) and its relative position in the fungal genome (up). (B) Confirmation of the gene disruption for the 7 CHSs by PCR in the mutants with PPT resistance and without a GFP signal. In each gene, D1 and D2 designate two different disruption mutants, and WT is the wild-type strain. Top panel: PCR conducted with the primers Bar-up and the confirmation primer CF2 of each gene, PCR products can be obtained only from the disruption mutants of the gene; Bottom panel: PCR conducted using primers CF1 and CF2; PCR products can be obtained only in WT strain. The positions of all primers are shown in (A). (C) Confirmation of the complementation of deletion mutants by PCR using the primers ORF-5 and ORF-3. C1 to C3: different complemented strains; WT: wild-type strain; D1: gene deletion mutant. The DNA ladder (DL 10004) on the left is purchased from Generay (Shanghai, China).

**
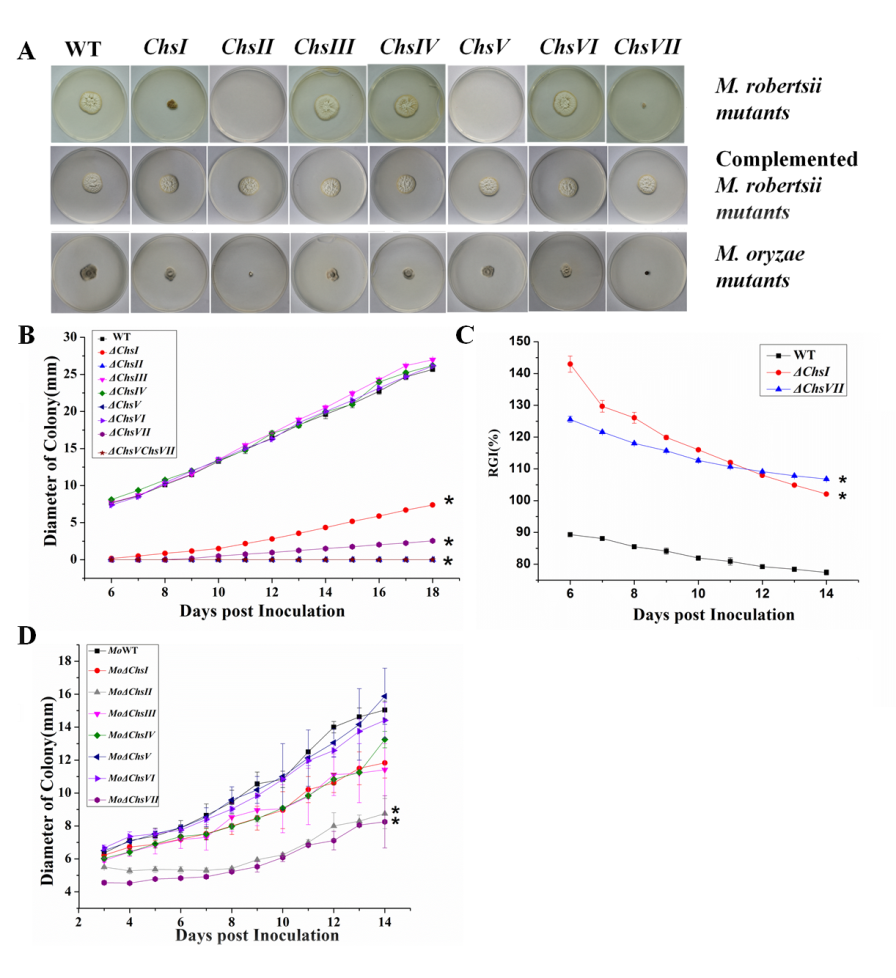
**

Fig. S4: Heat stress tolerance by *M. robertsii* and *M. oryzae* and their respective *Chs* mutants. (A) Heat stress tolerance of *M. robertsii* and *M. oryzae* on PDA plates; Growth curve under heat stress of *M. robertsii* *Chs* mutants (B), their respective complemented strains (C), and *M. oryzae Chs* mutants (D).

**
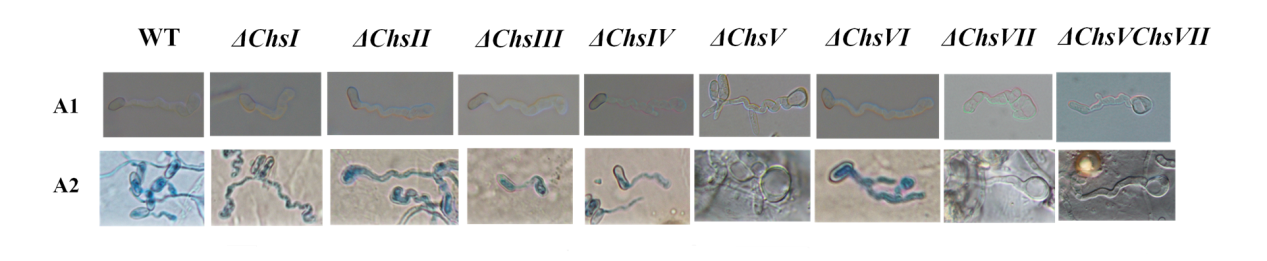
**

Fig. S5: appressorium formation by *M. robertsii Chs* mutants. Row A1: Appressoria formed on a hydrophobic surface; Row A2: locust hindwings.

**
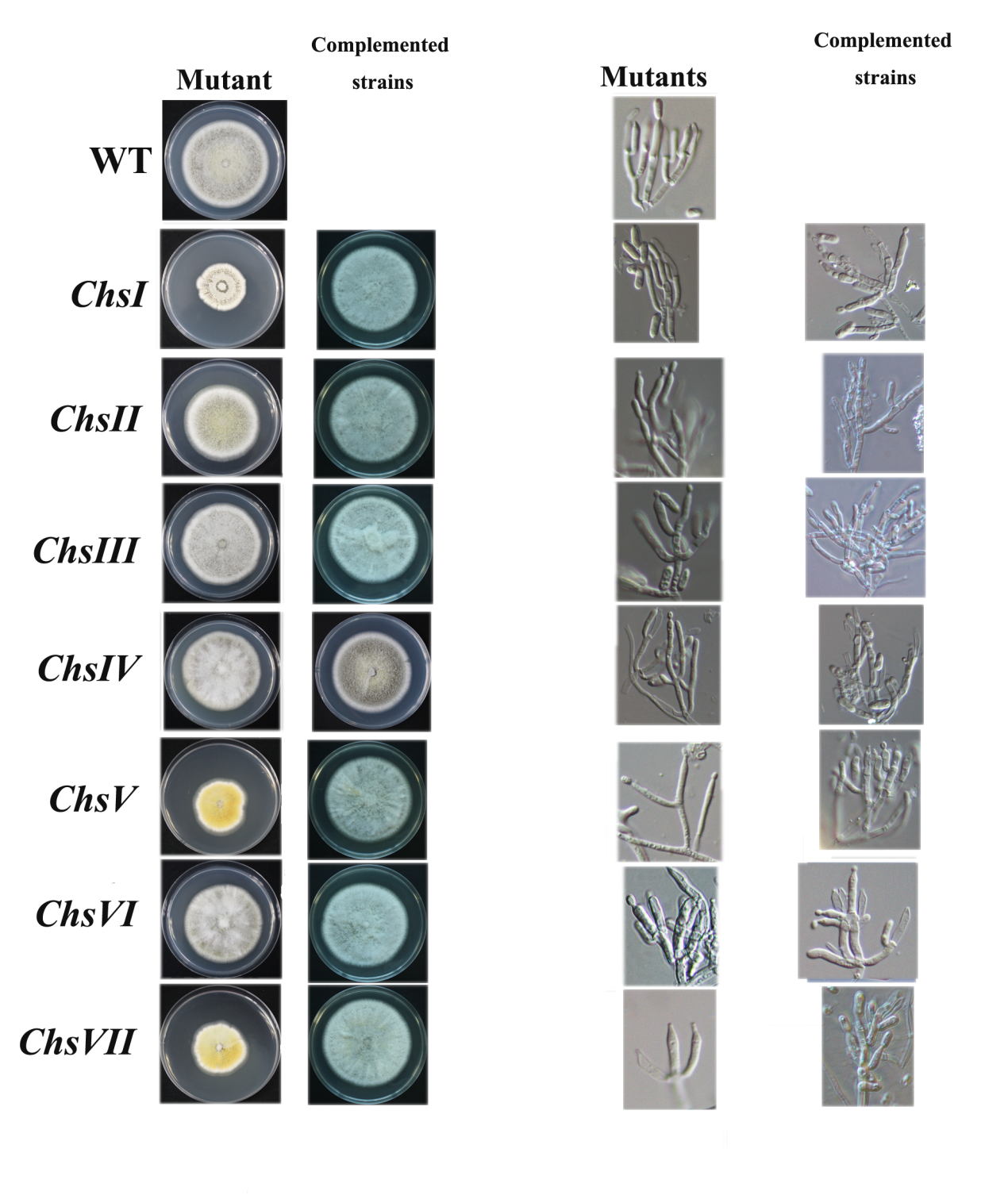
**

Fig. S6: Colony morphology (Left panel) and conidiophores (Right panel) of WT, *Chs* mutants and their respective complemented strains.
