# Supplementary material for: Evolution of the chitin synthase gene family correlates with fungal morphogenesis and adaption to ecological niches
Source: Sci Rep. 2017 Mar 16;7:44527. doi: 10.1038/srep44527 (PMC5353729; doi:10.1038/srep44527)
Supplement: Supplementary Table S11 [file srep44527-s12.doc]

**Supplementary Figures**

**Title**: Evolution of the chitin synthase gene family correlates with fungal morphogenesis and adaption to ecological niches

Ran Liu^ξ1^ (liuran1990@hotmail.com), Chuan Xu^ξ1^ (bioxc@zju.edu.cn), Qiangqiang Zhang^1^ (21407022@zju.edu.cn), Shiyi Wang^1^ (3140102621@zju.edu.cn), Weiguo Fang^*,1,2^ ([wfang1@zju.edu.cn](mailto:wfang1@zju.edu.cn))

1. Institute of Microbiology, College of Life Sciences, Zhejiang University, Hangzhou, 310058, Zhejiang, China
2. Institute of Insect Sciences, Zhejiang University, Hangzhou 310058, Zhejiang, China

*Corresponding author: Weiguo Fang

Tel: 86-571-88206668

E-mail: wfang1@zju.edu.cn

ξ: The authors contribute equally to this paper

Table S11. The characteristics of the 7 chitin synthases from *M. robertsii*

| Name | Protein ID (Genbank) | ORF (number of nucleotide) | Protein (number of amino acid) | Molecular Weight (kDa) | pI |
| --- | --- | --- | --- | --- | --- |
| ChsI | Maa_02999 | 2736 | 920 | 103.1 | 8.20 |
| ChsII | Maa_02740 | 2814 | 975 | 107.7 | 8.04 |
| ChsIII | Maa_03168 | 2685 | 894 | 101.2 | 7.64 |
| ChsIV | Maa_00137 | 3681 | 1226 | 136.7 | 8.88 |
| ChsV | Maa_01111 | 5355 | 1784 | 199.4 | 5.74 |
| ChsVI | Maa_01099 | 1911 | 710 | 727.9 | 8.28 |
| ChsVII | Maa_01112 | 5577 | 1858 | 205.6 | 7.01 |
