# Supplementary material for: Evolution of the chitin synthase gene family correlates with fungal morphogenesis and adaption to ecological niches
Source: Sci Rep. 2017 Mar 16;7:44527. doi: 10.1038/srep44527 (PMC5353729; doi:10.1038/srep44527)
Supplement: Supplementary Table S13 [file srep44527-s14.doc]

**Supplementary Figures**

**Title**: Evolution of the chitin synthase gene family correlates with fungal morphogenesis and adaption to ecological niches

Ran Liu^ξ1^ (liuran1990@hotmail.com), Chuan Xu^ξ1^ (bioxc@zju.edu.cn), Qiangqiang Zhang^1^ (21407022@zju.edu.cn), Shiyi Wang^1^ (3140102621@zju.edu.cn), Weiguo Fang^*,1,2^ ([wfang1@zju.edu.cn](mailto:wfang1@zju.edu.cn))

1. Institute of Microbiology, College of Life Sciences, Zhejiang University, Hangzhou, 310058, Zhejiang, China
2. Institute of Insect Sciences, Zhejiang University, Hangzhou 310058, Zhejiang, China

*Corresponding author: Weiguo Fang

Tel: 86-571-88206668

E-mail: wfang1@zju.edu.cn

ξ: The authors contribute equally to this paper

Table S13: Bioassay results^1^

| Fungal strains | Mean of LT_50_±SEM^2^ |
| --- | --- |
| WT | 8.8±0.29 |
| *ΔChsI* | 11.9±0.54 |
| *C-ΔChsI* | 9.13±0.32 |
| *ΔChsII* | 11.6±2.29 |
| *C-ΔChsII* | 9.23±0.24 |
| *ΔChsIII* | 10.9±0.91 |
| *C-ΔChsIII* | 9.04±0.68 |
| *ΔChsIV* | 11.6±2.18 |
| *C-ΔChsIV* | 9.21±0.72 |
| *ΔChsVI* | 9.1±2.11 |
| *C-ΔChsVI* | 9.4±0.47 |
| *C-ΔChsV* | 9.16±0.84 |
| *C-ΔChsVII* | 9.14±0.75 |

Note: 1: Inoculation dose was 1×10^7^ conidia/mL

2: No significant difference was identified among the fungal strains

LT_50_: the time needed to kill 50% of insects.
