# Supplementary material for: Evolution of the chitin synthase gene family correlates with fungal morphogenesis and adaption to ecological niches
Source: Sci Rep. 2017 Mar 16;7:44527. doi: 10.1038/srep44527 (PMC5353729; doi:10.1038/srep44527)
Supplement: Supplementary Table S17 [file srep44527-s18.doc]

**Supplementary Figures**

**Title**: Evolution of the chitin synthase gene family correlates with fungal morphogenesis and adaption to ecological niches

Ran Liu^ξ1^ (liuran1990@hotmail.com), Chuan Xu^ξ1^ (bioxc@zju.edu.cn), Qiangqiang Zhang^1^ (21407022@zju.edu.cn), Shiyi Wang^1^ (3140102621@zju.edu.cn), Weiguo Fang^*,1,2^ ([wfang1@zju.edu.cn](mailto:wfang1@zju.edu.cn))

1. Institute of Microbiology, College of Life Sciences, Zhejiang University, Hangzhou, 310058, Zhejiang, China
2. Institute of Insect Sciences, Zhejiang University, Hangzhou 310058, Zhejiang, China

*Corresponding author: Weiguo Fang

Tel: 86-571-88206668

E-mail: wfang1@zju.edu.cn

ξ: The authors contribute equally to this paper

Table S17 Primers used in this study

| Primer | Sequence | Usage |
| --- | --- | --- |
| ∆ChsI-5-1 | GGGGACAGCTTTCTTGTACAAAGTGGAAGTACTCCGTAGACATCTC | Disruption of *ChsI* |
| ∆ChsI-5-2 | GGGGACTGCTTTTTTGTACAAACTTGTGACGTGACTATTCGGTAG |
| ∆ChsI-3-1 | GGGGACAACTTTGTATAGAAAAGTTGTTACTCGTTTGGTATAGGAG |
| ∆ChsI-3-2 | GGGGACAACTTTGTATAATAAAGTTGTTCAGGCTCAATAGTCAGG |
| ∆ChsI-CF-1 | ATCGAGAAGAAGGATGTG | Confirmation of the Disruption of  *ChsI* |
| ∆ChsI-CF-2 | TCTCTGCACCTTACTTTC |
| C-∆ChsI-5 | ATTTAAATTTACTCCCATACGACGAC | Cloning the genomic clone of  *ChsI* for complementation |
| C-∆ChsI-3 | ATTTAAATTTTAACATCGTCCATAC |
| C-∆ChsI-ORF-5 | CACGATGAGGAAGAGAG | Confirmation of the  complementation of *ChsI* |
| C-∆ChsI-ORF-3 | AAGTGAGTACAGAGAAG |
| ∆ChsII-5-1 | GGGGACAGCTTTCTTGTACAAAGTGGAATCCCCAATGCTGATTTCC | Disruption of *ChsII* |
| ∆ChsII-5-2 | GGGGACTGCTTTTTTGTACAAACTTGTAATGCGGCTCTTGTTAG |
| ∆ChsII-3-1 | GGGGACAACTTTGTATAGAAAAGTTGTTACTCCCTCGTTCAATTTC |
| ∆ChsII-3-2 | GGGGACAACTTTGTATAATAAAGTTGTATGACCCATGCCGATTCC |
| ∆ChsII-CF-1 | TCACTCAAATGCTCTAAC | Confirmation of the Disruption of |
| ∆ChsII-CF-2 | ATGCCCACTGTCATGTCC | *ChsII* |
| C-∆ChsII-5 | ggGCTAGCATACTGCCAATTAGATG | Cloning the genomic clone of |
| C-∆ChsII-3 | ggTTTAAATGTCCGTTACCATTAAC | *ChsII* for complementation |
| C-∆ChsII-ORF-5 | TCCAGAATACACACTAC | Confirmation of the |
| C-∆ChsII-ORF-3 | TTCAAGCAGAATATCAG | complementation of *ChsII* |
| ∆ChsIII-5-1 | GGGGACAGCTTTCTTGTACAAAGTGGAAAAAGGATCAATCGTTCGG | Disruption of *ChsIII* |
| ∆ChsIII-5-2 | GGGGACTGCTTTTTTGTACAAACTTGTGTACGAATGTTAGATTGG |
| ∆ChsIII-3-1 | GGGGACAACTTTGTATAGAAAAGTTGTTGTCTTCTACACGCTCATC |
| ∆ChsIII-3-2 | GGGGACAACTTTGTATAATAAAGTTGTTACAAGCTGGTTGCTAGG |
| ∆ChsIII-CF-1 | TTGCTGCCATCTTCATTG | Confirmation of the Disruption of |
| ∆ChsIII-CF-2 | GCAAAACGCGGACAGTGG | *ChsIII* |
| C-∆ChsIII-5 | ggTCTAGAGTTCAGAGAAGCCTCATC | Cloning the genomic clone of |
| C-∆ChsIII-3 | ggATTTAAATAATCAAACTCCGTGTTC | *ChsIII* for complementation |
| C-∆ChsIII-ORF-5 | CCAGCCATTGATACAAG | Confirmation of the |
| C-∆ChsIII-ORF-3 | TCCATTTAGCCATCGAC | complementation of *ChsIII* |
| ∆ChsIV-5-1 | GGGGACAGCTTTCTTGTACAAAGTGGAATAGCACCACCTGGCAAC | Disruption of *ChsIV* |
| ∆ChsIV-5-2 | GGGGACTGCTTTTTTGTACAAACTTGTAGGTGCCTACTTACCAAG |
| ∆ChsIV-3-1 | GGGGACAACTTTGTATAGAAAAGTTGTTTTGACGAGCGAATGACAG |
| ∆ChsIV-3-2 | GGGGACAACTTTGTATAATAAAGTTGTGAGTTATTGCTGTTGCTG |
| ∆ChsIV-CF-1 | GATGCTGATTGTCAAGTG | Confirmation of the Disruption of |
| ∆ChsIV-CF-2 | CGTTCGTGTCATATTTG | *ChsIV* |
| C-∆ChsIV-5 | ggTCTAGATGTATGAGTCAGGATTC | Cloning the genomic clone of |
| C-∆ChsIV-3 | ggATTTAAATACTGATTCAAGTGGAAG | *ChsIV* for complementation |
| C-∆ChsIV-ORF-5 | AGTCTGGCTCCAGGTTC | Confirmation of the |
| C-∆ChsIV-ORF-3 | TGTAATGACGATGAGTG | complementation of *ChsIV* |
| ∆ChsV-5-1 | GGGGACAGCTTTCTTGTACAAAGTGGAAGTTCCCGCAGAGACAGAC | Disruption of *ChsV* |
| ∆ChsV-5-2 | GGGGACTGCTTTTTTGTACAAACTTGTAGTGCTTGGATGCAAAGG |
| ∆ChsV-3-1 | GGGGACAACTTTGTATAGAAAAGTTGTTACCGACGCAATAGAAACC |
| ∆ChsV-3-2 | GGGGACAACTTTGTATAATAAAGTTGTGCGTCCTTGTATACATCC |
| ∆ChsV-CF-1 | GCAACTTGCTGCTCTACC | Confirmation of the Disruption of |
| ∆ChsV-CF-2 | GAGTTCTGAATGACTTGG | *ChsV* |
| C-∆ChsV-5 | ccCCCGGGTTACCACTGGCAATATG | Cloning the genomic clone of |
| C-∆ChsV-3 | ggATTTAAATTTGTCCAAGACTCTAAC | *ChsV* for complementation |
| C-∆ChsV-ORF-5 | TGAATGCGATACATAAC | Confirmation of the |
| C-∆ChsV-ORF-3 | CACATTGACACCATCTTC | complementation of *ChsV* |
| ∆ChsVI-5-1 | GGGGACAGCTTTCTTGTACAAAGTGGAAACTGGATCAGAGAGCAGG | Disruption of *ChsVI* |
| ∆ChsVI-5-2 | GGGGACTGCTTTTTTGTACAAACTTGTGATATCCAAGTTAGTTCC |
| ∆ChsVI-3-1 | GGGGACAACTTTGTATAGAAAAGTTGTTCGCAAGCGAAGTGTTGTG |
| ∆ChsVI-3-2 | GGGGACAACTTTGTATAATAAAGTTGTCCTCTCAATGAGCATATG |
| ∆ChsVI-CF-1 | AGAAGCTGCGGAGCAGG | Confirmation of the Disruption of |
| ∆ChsVI-CF-2 | TGTGATAGCTTGTGATGG | *ChsVI* |
| C-∆ChsVI-5 | ggGCTAGCCTGCCATCTGATTCTAC | Cloning the genomic clone of |
| C-∆ChsVI-3 | ccCCCGGGCAACAGAAGTTCTCCTG | *ChsVI* for complementation |
| C-∆ChsVI-ORF-5 | GTTCTGGATGATTACAC | Confirmation of the |
| C-∆ChsVI-ORF-3 | CCTTGGTTCTCACGAAC | complementation of *ChsVI* |
| ∆ChsVII-5-1 | GGGGACAGCTTTCTTGTACAAAGTGGAAGGCAGTGCAACTTATACGG | Disruption of *ChsVII* |
| ∆ChsVII-5-2 | GGGGACTGCTTTTTTGTACAAACTTGTAGGATTTGGAGTGAAGAG |
| ∆ChsVII-3-1 | GGGGACAACTTTGTATAGAAAAGTTGTTCAAGACGTCCTCCAAACC |
| ∆ChsVII-3-2 | GGGGACAACTTTGTATAATAAAGTTGTAGCAGAGACTTGTACAGG |
| ∆ChsVII-CF-1 | GGAAGCAACTTCTATAC | Confirmation of the Disruption of |
| ∆ChsVII-CF-2 | AATTGATCAAGGCTGGTG | *ChsVII* |
| C-∆ChsVII-5 | CCCGGGTGTTTGCTCAGGCTTAC | Cloning the genomic clone of |
| C-∆ChsVII-3 | CCCGGGTTTCTTTCGTTCGCTAC | *ChsVII* for complementation |
| C-∆ChsVII-ORF-5 | GTGCCTGGATAAGACTC | Confirmation of the |
| C-∆ChsVII-ORF-3 | ACCTCTTCCGACTCCAAC | complementation of *ChsVII* |
| ∆ChsVChsVII-5-1 | GGGGACAGCTTTCTTGTACAAAGTGGAAGTACTCCGTAGACATCTC | Disruption of *ChsVChsVII* |
| ∆ChsVChsVII-5-2 | GGGGACTGCTTTTTTGTACAAACTTGTCTCTTCACCAATCAACTC |
| ∆ChsVChsVII-3-1 | GGGGACAACTTTGTATAGAAAAGTTGTTGTGCCTGGATAAGACTC |
| ∆ChsVChsVII-3-2 | GGGGACAACTTTGTATAATAAAGTTGTATCAGTCTTGTTGCGTC |
| ∆ChsVChsVII-CF-1 | ATCCGACACGCACATTAC | Confirmation of the Disruption of |
| ∆ChsVChsVII-CF-2 | TCTGACGGTGAAGGATG | *ChsVChsVII* |
